# Genome-Wide Identification of the *SiNHX* Gene Family in Foxtail Millet (*Setaria Italica*) and Functional Characterization of *SiNHX7* in *Arabidopsis*

**DOI:** 10.3390/ijms26157139

**Published:** 2025-07-24

**Authors:** Xiaoqian Chu, Dan-Ying Chen, Mengmeng Sun, Jiajing Zhang, Minghua Zhang, Hejing Wu, Hongzhi Wang, Shuqi Dong, Xiangyang Yuan, Xiaorui Li, Lulu Gao, Guanghui Yang, Jia-Gang Wang

**Affiliations:** 1 Special Orphan Crops Research Center of the Loess Plateau, Ministry of Agriculture and Rural Affairs, College of Agriculture, Shanxi Agricultural University, Taigu 030801, China; chuxiaoqian@sxau.edu.cn (X.C.); c1178381281@163.com (D.-Y.C.); m19834545306@163.com (M.S.); zjj13546153480@163.com (J.Z.); 17806709769@163.com (M.Z.); pangkai19990921@163.com (H.W.); 19821282073@163.com (H.W.); dongshuqi@sxau.edu.cn (S.D.); yuanxiangyang200@sxau.edu.cn (X.Y.); lixiaorui@sxau.edu.cn (X.L.); lulugao2013@126.com (L.G.); yanggh1991@163.com (G.Y.); 2 HouJi Laboratory in Shanxi Province, Shanxi Agricultural University, Taigu 030801, China

**Keywords:** foxtail millet, *SiNHX* gene family, *SiNHX7*, salt stress

## Abstract

Plant growth is susceptible to abiotic stresses like salt and drought, and Na^+^/H^+^ antiporters (NHXs) play a pivotal role in stress responses. NHX proteins belong to the CPAs (cation/proton antiporters) family with a conserved Na^+^ (K^+^)/H^+^ exchange domain, which is widely involved in plant growth, development, and defense. While *NHX* genes have been extensively studied in model plants (e.g., *Arabidopsis thaliana* and *Oryza sativa*), research in other species remains limited. In this study, we identified nine *NHX* genes in foxtail millet (*Setaria italica*) and analyzed their systematic phylogeny, gene structure, protein characteristics, distribution of the chromosome, collinearity relationship, and cis-elements prediction at the promoter region. Phylogenetic analysis revealed that the members of the *SiNHX* gene family were divided into four subgroups. RT-qPCR analysis of the *SiNHX* family members showed that most genes were highly expressed in roots of foxtail millet, and their transcriptional levels responded to salt stress treatment. To determine *SiNHX7*’s function, we constructed overexpression *Arabidopsis* lines for each of the two transcripts of *SiNHX7*, and found that the overexpressed plants exhibited salt tolerance. These findings provide valuable insights for further study of the function of *SiNHX* genes and are of great significance for breeding new varieties of salt-resistant foxtail millet.

## 1. Introduction

One of the direct impacts of climate change is the alteration of precipitation and evaporation patterns, which results in increased soil salinity and salinization. Salt stress is a critical environmental factor influencing plant growth and crop yield, and it also poses a significant challenge to crop production and ecological environment construction [1]. Unfavorable growth conditions can affect plant growth, development, and yield [2]. Salt stress causes a series of damages to plants, including osmotic stress, oxidative stress, and ion toxicity [3,4,5], which in turn affect plant growth and development, leading to reduced agricultural yield and damaged ecosystems [6,7]. To adapt to salt stress, plants have evolved various physiological and biochemical mechanisms for resistance [8], such as re-establishing ion balance, increasing osmotic protective substances, increasing antioxidant substances, and increasing antioxidant activity [9,10]. Among these, the re-establishment of ion homeostasis and maintenance of ion balance represent critical strategies for plants to combat salt stress [11,12]. Plants can maintain the cellular content of Na^+^ and K^+^ within an optimal range through a “preserving K^+^ and excreting Na^+^” mechanism, thereby alleviating salt stress-induced cellular damage [10,13,14].

Maintaining intracellular ion and pH homeostasis under abiotic stress is crucial for plant resistance to stressed environments [9,15,16]. The Na^+^ (K^+^)/H^+^ transporter is an ion antiporter located in the vacuole membrane, and it is a kind of transmembrane transport protein commonly found in biology responsible for Na^+^ (K^+^)/H^+^ exchange. The Salt Overly Sensitive (SOS) signaling transduction pathway is mainly responsible for excreting Na^+^ from root cells, and it is involved in maintaining the dynamic balance of plant ions, which is closely associated with plants’ salt tolerance [17,18,19]. The expression of Na^+^ (K^+^)/H^+^ antiporters is regulated by NHX family genes. NHX proteins contain the Na^+^/H^+^ exchange (PF00999) domain, which can maintain ion homeostasis by mediating Na^+^/H^+^ or K^+^/H^+^ exchange, and play a critical role in diverse physiological and metabolic processes in plants [20], such as maintaining ion homeostasis [21,22,23], regulating pH [24,25], affecting vesicle transport [24], and regulating plant growth and development [26]. NHX is highly conserved across all eukaryotes [27]. The first *NHX* gene cloned was barley (*Hordeum vulgare*) *NHX1* [28], and subsequently identified in *Arabidopsis thaliana* [29,30,31]. *Arabidopsis thaliana* contains eight NHX family members, which are classified into three categories based on their localization: AtNHX7 and AtNHX8 are localized to the plasma membrane; AtNHX1-AtNHX4 are vacuole-localized; and AtNHX5 and AtNHX6 are located in vesicles [32].

Studies have demonstrated that NHX plays a crucial role in plant resistance to abiotic stress [33], acting as a key factor in plant salt tolerance. Overexpression of *NHX* family genes can enhance the activity of Na^+^ (K^+^)/H^+^ antiporters in plants, thereby regulating cytoplasmic pH, maintaining Na^+^ (K^+^) concentration, K^+^/Na^+^ ratio, maintaining cell turgor, and controlling cell expansion [34,35]. Overexpression of *AtNHX1* and its homologues in *Arabidopsis thaliana*, rice (*Oryza sativa*), and peanut (*Arachis hypogaea*) can improve salt tolerance [36,37,38]. Overexpression of *HtNHX1* or *HtNHX2* in rice enhances the plants’ salt tolerance. Moreover, *HtNHX2* transgenic rice can maintain high yield under low potassium or nutrient deficiency conditions, which has great application potential [39]. Under salt–alkali conditions, *Arabidopsis* overexpressing *GmNHX6* can maintain a low Na^+^/K^+^ ratio, thereby enhancing its salt tolerance [40].

In addition to their function in maintaining pH and ion homeostasis, NHX proteins are involved in various physiological and biochemical processes, including regulating cell shape and volume, vesicle transport, protein sorting, cell stress response, and growth and development [21,22,32,41,42,43]. In *Arabidopsis thaliana*, NHX5 and NHX6 affect vacuole pH and transmembrane protein sorting, and the *nhx5 nhx6* double-knockout mutant exhibits smaller rosette leaves, shorter plants, delayed flowering and bolting, reduced seed yield, and disrupted vacuole K^+^ and pH homeostasis [21]. Growth and development of the *Arabidopsis thaliana nhx1 nhx2* double knockout mutant were inhibited, characterized by shorter hypocotyls and abnormal fruit development, with lower vacuole pH and K^+^ contents compared to the wild type, and exogenous Na^+^ application can partially restore the phenotype [22,41]. NHX1 and NHX2 can also regulate cell turgor by modulating the uptake of K^+^, thereby influencing the growth and development of stomatal guard cells [41].

Foxtail millet (*Setaria italica*), one of the world’s oldest domesticated crops, is primarily cultivated in Asia and also grown in arid and semi-arid regions across Eurasia and Africa [44,45,46]. Renowned for its remarkable traits, it exhibits drought resistance, tolerance to infertile soil, wide adaptability, and rich nutritional value [47,48]. Research on the growth, development, and salt tolerance mechanisms of foxtail millet not only facilitates the efficient exploitation and utilization of saline–alkali land, bearing significant implications for agricultural production and practical applications, but also plays a pivotal role in deciphering plant salt signaling pathways.

In this study, we performed a genome-wide identification of the *SiNHX* gene family in foxtail millet (*Setaria italica*). Further analyses included phylogenetic tree construction, conserved protein motif identification, gene structure analysis, cis-element analysis, distribution of the chromosome, and collinearity analysis. Additionally, expression profiles of *SiNHX* gene family members across different tissues, organs, and various developmental stages were investigated, revealing that most members exhibit relatively high expression levels in roots. Finally, through the functional study of two transcripts of *SiNHX7*, the overexpression of *SiNHX7a* and *SiNHX7b* enhanced, to a certain extent, the growth and development of the vegetative organs of transgenic *Arabidopsis* and conferred salt stress tolerance. The findings of this study provide valuable insights for a deeper understanding of the *SiNHX* gene family and further promote the functional study of *SiNHX* genes.

## 2. Results

### 2.1. Phylogenetic and Structural Characterization of NHX Gene Family in Foxtail Millet

**Figure 1:**
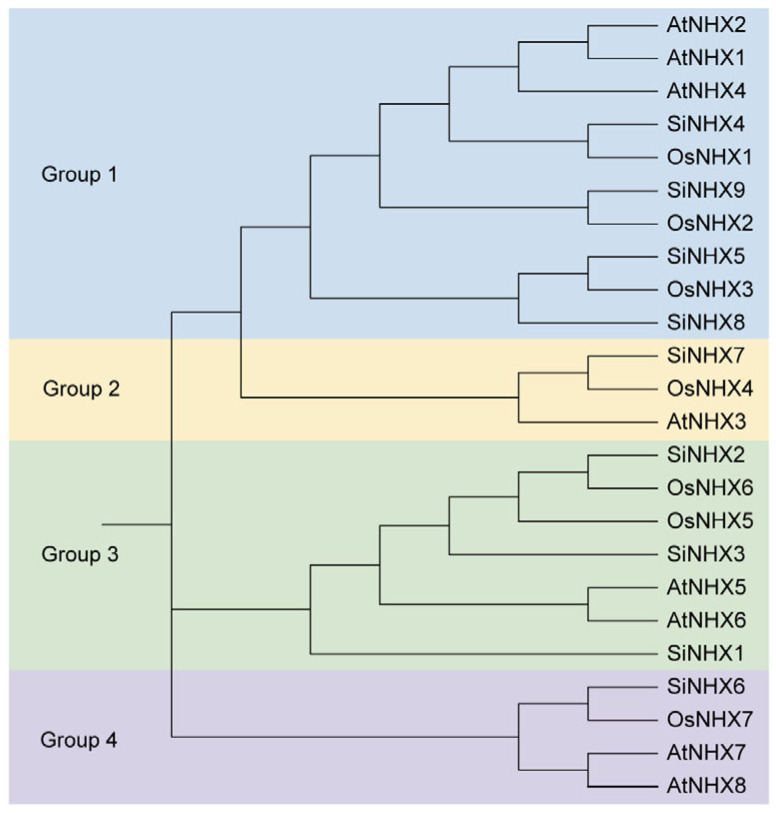
Phylogenetic tree depicting NHX proteins from foxtail millet, rice, and *Arabidopsis*. The Neighbor Joining method in MEGA 11 was utilized to construct the phylogenetic tree, where various colored branches represent different groups. For the analysis, full-length protein sequences of the NHX family were aligned, ensuring a comprehensive comparison. The corresponding gene IDs can be found in Supplementary Table S1.

A total of nine *SiNHXs* were identified after removing duplicate genes from the Yugu1 genome. To gain deeper insights into the evolutionary relationships between foxtail millet and the classical model plant *Arabidopsis* and the gramineous model plant rice (*Oryza sativa*), we compared foxtail millet with rice and *Arabidopsis NHX* gene family members. The phylogenetic tree was constructed using the NHX protein sequences of *Arabidopsis*, rice, and foxtail millet. The results showed that all NHX members were divided into four groups, among which Group 2 had the smallest members and Group 1 had the largest 10 members. Moreover, in most instances, the homologous groups exhibited monophyletic relationships, with foxtail millet NHX members clustering more closely with rice NHX proteins than *Arabidopsis.* This indicates that foxtail millet and rice share a closer evolutionary relationship, as they both belong to the Poaceae (Figure 1).

**Figure 2:**
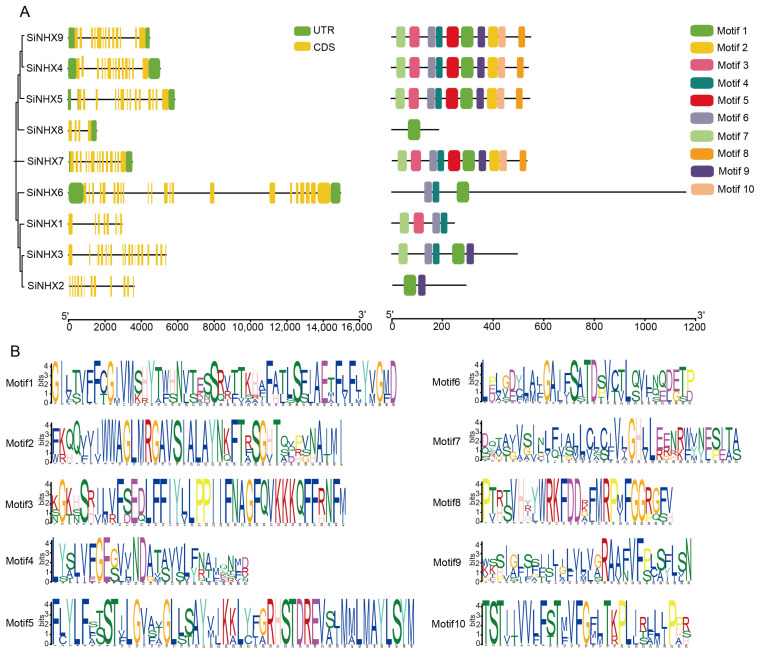
DNA structures and conserved protein motifs of SiNHXs. (**A**) The DNA structures and conserved protein motifs of the *SiNHXs* were identified and illustrated using TBtools. Colored rectangles represent different conserved motifs or exons and introns in protein sequences or DNA sequences, respectively. (**B**) The consensus-conserved motifs of SiNHX proteins were identified in the MEME suite web server.

To gain a clearer and more intuitive understanding of the evolutionary relationships among *SiNHX* members, we visualized their gene structures and motifs. The results showed that three genes (*SiNHX1*, *SiNHX2,* and *SiNHX3*) lacked untranslated regions (UTRs), *SiNHX8* lacked 5′ UTR Regions, and the remaining five genes contained both 5′ and 3′ UTRs (Figure 2A). Analysis of gene structure revealed variations in the length of DNA sequences, as well as intron and exon counts, across members of various groups and among different individuals within the same group. Among *SiNHX* members, *SiNHX1* possesses the least number of exons (8) and introns (7), while *SiNHX6* contains the most exons (22) and introns (21). A conserved motif analysis identified 10 conserved motifs among the nine SiNHX proteins (Figure 2B). Generally, these motifs were evenly distributed, and SiNHX proteins within the same subgroup shared similar motif compositions and arrangements.

### 2.2. Analysis of Putative Cis-Acting Regulatory Elements (CAREs or Cis-Elements) in SiNHX Genes

**Figure 3:**
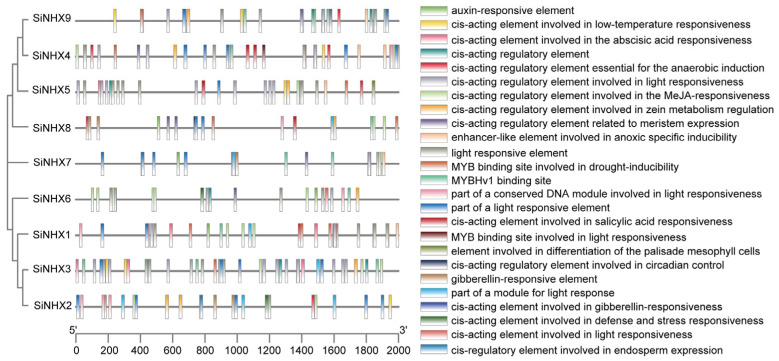
Cis-element analysis of promoters of *SiNHX* genes. Cis-elements with various regulatory functions were depicted on the 2000 bp promoter region of *SiNHX* genes, with their positions indicated. A 2000 bp promoter sequence is represented by a dark line, while rectangles in different colors denote various types of cis-elements.

To further explore the function of the *SiNHX* genes, we searched the promoter region 2000 bp upstream of the transcription start site of the *SiNHX* genes in the Plant Promoter database (PlantCARE). Analysis of the cis-acting elements located in the promoter region of the *SiNHXs* gene revealed the presence of three principal types of cis-acting elements within its promoter sequence (Figure 3). The first category involves plant hormone-responsive elements, including gibberellin (GA), methyl jasmonate (MeJA), auxin (IAA), and abscisic acid (ABA). The second category is related to stress responses, including anaerobic induction, defense responses, light responses, and low temperature responses. The last category relates to the regulation of plant growth and development, which involves meristem expression regulation, circadian rhythm regulation, endosperm expression regulation, zein metabolism regulation, MYB or MYBHv1 binding sites, etc. Notably, the presence of light-responsive and phytohormone-responsive cis-elements suggests that the *SiNHX* gene family is primarily involved in photoresponse processes (e.g., photosynthesis-based or circadian-regulated photoresponse), as well as phytohormone-regulated biological processes and responses. Nevertheless, the diversity in the number of different cis-elements across various promoters highlights variability in the regulatory mechanisms controlling *SiNHX* gene expression. These results imply that *SiNHXs* may modulate plant changes throughout the life cycle by affecting a series of processes such as hormone signaling responses, stress responses, and the regulation of growth and development.

### 2.3. Chromosome Distribution and Collinearity Analysis of SiNHX Gene Family Members

**Figure 4:**
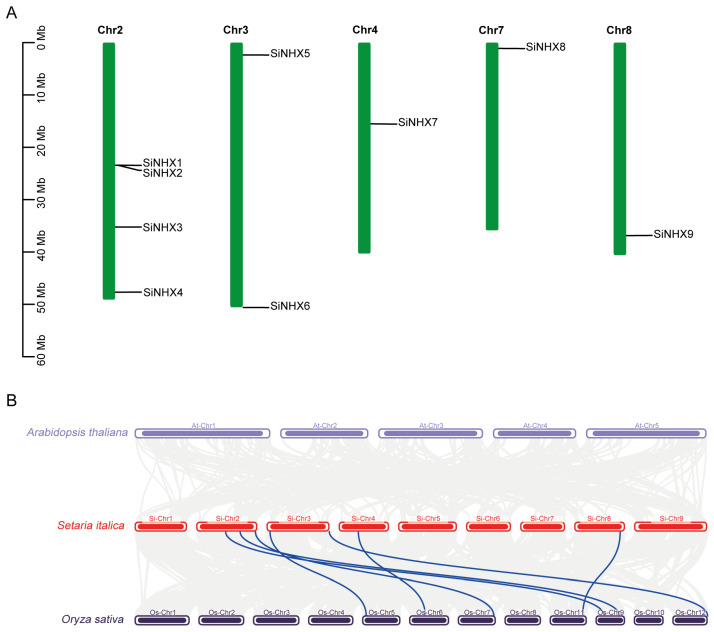
Chromosomal distribution of *SiNHX* genes and collinearity analysis of *NHX* genes among three different species. (**A**) The distribution of 9 *NHX* genes across 5 chromosomes in foxtail millet. (**B**) Result of colinearity analysis for *NHX* genes in three species: *Setaria italica*, *Arabidopsis thaliana*, and *Oryza sativa*. Collinear relationships between *NHX* genes of different species are highlighted by blue lines.

To reveal the chromosomal distribution of *SiNHX* genes, we mapped the physical locations of each *SiNHX* gene onto the nine chromosomes of foxtail millet. The findings indicated that *SiNHX* genes were distributed across five of the nine chromosomes, exhibiting uneven distribution. Four *SiNHX* members were identified on the second chromosome. No *SiNHX* was found on chromosomes 1, 5, 6, and 9 (Figure 4A). In addition, to understand the collinearity of *NHX* genes among different species, we performed a collinearity analysis of *NHX* genes in three representative species: foxtail millet (*Setaria italica*), the dicot model plant *Arabidopsis thaliana*, and the Poaceae model plant rice (*Oryza sativa*) (Figure 4B). The results showed no collinearity between foxtail millet and *Arabidopsis*. In contrast, most *NHX* genes in foxtail millet exhibited multiple homologous counterparts with irregular chromosomal distributions in rice, suggesting a closer evolutionary association between the two Poaceae species.

### 2.4. Physical and Chemical Properties of NHX Proteins in Foxtail Millet

Analysis of the physical and chemical characteristics of SiNHX proteins in foxtail millet revealed that SiNHX1 had the lowest isoelectric point (pI), at 5.26, while SiNHX8 exhibited the highest (pI = 9.87). The pI < 7 (SiNHX1, SiNHX2, SiNHX3, and SiNHX6) were classified as acidic, while the remaining five were basic (pI > 7). The aliphatic index ranged from 64.54 (SiNHX8) to 115.68 (SiNHX5), with an average of 101.64, indicating that the thermostability among SiNHX proteins was relatively consistent. Among all the proteins, the Grand Average of Hydropathicity (GRAVY) of SiNHX1 is the largest, which can reach 0.821, and the average value of SiNHX8 is the smallest, which is only −0.514. According to the general principle that a GRAVY value between −0.5 and 0.5 indicates amphoteric proteins, positive values denote hydrophobic proteins, and negative values signify hydrophilic proteins, 5 out of 9 SiNHX proteins are classified as hydrophobic. Among all members, only SiNHX2, SiNHX3, and SiNHX6 have amphoteric characteristics (GRAVY: −0.5 to 0.5). The instability index (II) of the proteins ranged from 31.02 (SiNHX5) to 49.12 (SiNHX1). Notably, four proteins (SiNHX4, SiNHX5, SiNHX7, and SiNHX9) had an instability index below 40, meeting the criterion for stable proteins, whereas the remaining five proteins (SiNHX1, SiNHX2, SiNHX3, SiNHX6, and SiNHX8) exceeded this threshold (Table 1).

### 2.5. Prediction Secondary Structure and Subcellular Localization of SiNHX Proteins

Secondary structure predictions indicated that all SiNHX proteins contain α-helices, extended strands, and random coils. Subcellular localization prediction showed that among the nine SiNHX proteins, all of them were localized to vacuoles except SiNHX6, which was localized in the cell membrane (Table 2).

### 2.6. Tissue Expression Analysis of the SiNHX Gene Family

**Figure 5:**
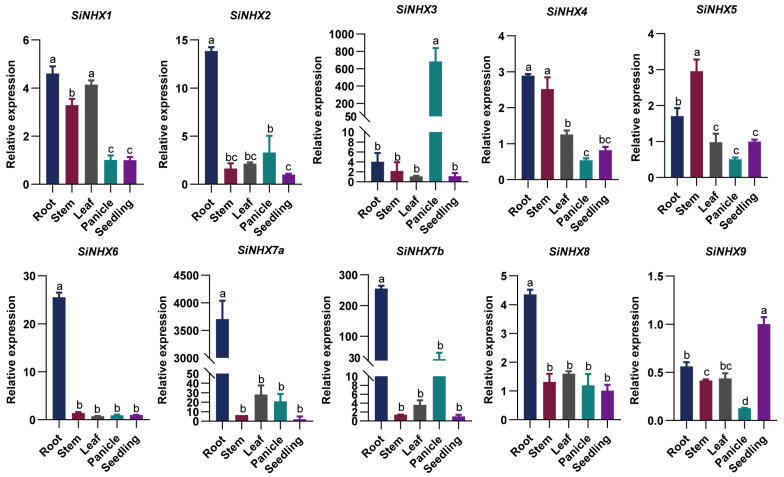
Expression of *SiNHX* gene family members in different tissues of JG21. Significant differences are analyzed using ordinary one-way ANOVA by Tukey’s method; lowercase letters indicate statistical significance at *p* < 0.05.

In the process of identifying members of the *SiNHX* gene family, we discovered that *SiNHX7* produces two transcripts (designated *SiNHX7a* and *SiNHX7b*). Sequence analysis of the two transcripts showed that while their genomic sequences are identical, their coding sequences (CDS) and deduced amino acid sequences differ. Predictions of the protein transmembrane domains and functional domains revealed that SiNHX7a contains more domains, which may be attributed to its longer amino acid sequence. Therefore, we designed specific primers for the two transcripts (Supplementary Figure S1, Supplementary Table S2). The expression profiling of *SiNHX* genes in foxtail millet can be categorized into two main groups. A group consisting of *SiNHX1*, *SiNHX2*, *SiNHX4*, *SiNHX6*, *SiNHX7*, and *SiNHX8* exhibited higher transcript abundances in roots relative to other tissues, which implies that most members of the *SiNHX* gene family are functionally associated with plant resistance to salt stress. In contrast, another group, including *SiNHX3*, *SiNHX5*, and *SiNHX9*, showed preferential expression in non-root tissues: *SiNHX3* was highly expressed in panicles with levels significantly exceeding those in other tissues; *SiNHX5* displayed elevated expression in stems; and *SiNHX9* was most abundant in 7-day-old seedlings (Figure 5).

### 2.7. Analysis of Root Expression of SiNHX Gene Family Treated with Salt Stress

**Figure 6:**
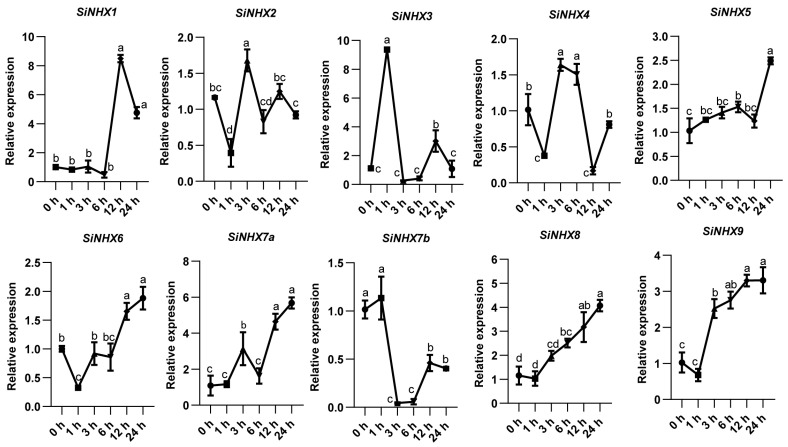
Analysis of expression of the *SiNHX* gene family in roots treated with salt stress. Significant differences are analyzed using ordinary one-way ANOVA by Tukey’s method; lowercase letters indicate statistical significance at *p* < 0.05.

Seven-day-old JG21 foxtail millet seedlings were treated with 150 mM NaCl for 0 h, 1 h, 3 h, 6 h, 12 h, and 24 h, and temporal expression patterns of *SiNHX* genes in roots were analyzed. All family members exhibited dynamic expression changes in response to salt treatment duration (Figure 6). Specifically, *SiNHX8* and *SiNHX9* showed decreased expression at 1 h post-stress, followed by continuous upregulation with prolonged treatment. *SiNHX1* displayed no significant change within the first 6 h but a marked increase at 12 h, and *SiNHX2* exhibited fluctuating expression patterns over time, potentially reflecting their distinct functional roles in salt stress responses.

### 2.8. Overexpression of SiNHX7 Enhances Salt Tolerance of Transgenic Arabidopsis

Based on the foregoing data, we identified that *SiNHX7* produces two transcripts, both of which exhibit remarkably high expression levels in roots. Given their distinct transcriptional response patterns to salt stress, we selected *SiNHX7* as the focus of subsequent investigations. Seed germination is a pivotal initial stage in plant growth and development, with its success directly influencing subsequent growth and survival. Under salt stress conditions, seed germination becomes a formidable challenge, as it serves as a critical determinant of plant salt tolerance. As depicted in Supplementary Figure S2, when germinated on NaCl-containing media, wild-type (WT) *Arabidopsis* seeds exhibited a significantly lower germination rate compared to *SiNHX7-OX* transgenic seeds. The inhibitory effects of salt stress severely hindered radicle and plumule development, preventing many WT seeds from breaking through the seed coat. In contrast, *SiNHX7* overexpression effectively mitigated these inhibitory effects, enabling transgenic seeds to establish a robust foundation for subsequent growth.

**Figure 7:**
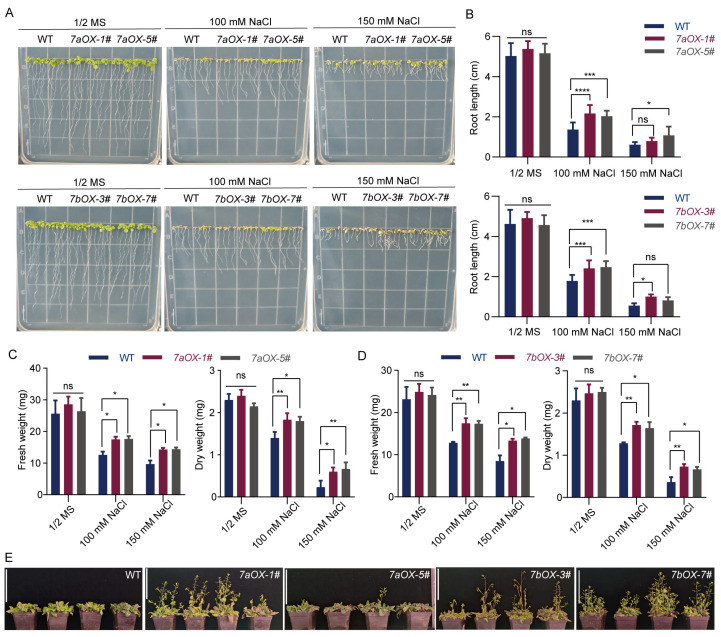
Phenotypic analysis of wild-type (WT) and *SiNHX7-OX* transgenic *Arabidopsis* lines under salt stress. (**A**) Root growth of WT and *SiNHX7-OX* transgenic seedlings vertically grown on NaCl-containing 1/2 MS medium plates. Scale bar = 1 cm. (**B**) Main root lengths of WT and *SiNHX7-OX* transgenic *Arabidopsis* lines measured 10 days after transfer to plates with NaCl. Data are presented as mean ± SD (*n* = 30). Significant differences were analyzed by two-way ANOVA followed by Tukey’s multiple comparison test (ns: no significance; * *p* < 0.05; *** *p* < 0.001; **** *p* < 0.0001). (**C**,**D**) Fresh weight and dry weight of WT and *SiNHX7-OX* transgenic *Arabidopsis* lines measured 10 days after plate transfer. Total weight of 10 plants was measured in triplicate. Data are presented as mean ± SD (*n* = 3). Significant differences were performed using two-way ANOVA followed by Tukey’s multiple comparison test (ns: no significance; * *p* < 0.05; ** *p* < 0.01). (**E**) Salt tolerance phenotypes of WT and *SiNHX7-OX* transgenic seedlings grown in soil. Scale bar = 10 cm.

Root growth plays a vital role in plant survival and development under salt stress. As shown in Figure 7A, WT *Arabidopsis* seedlings grown vertically on NaCl-containing media displayed pronounced growth retardation, characterized by stunted primary root development and sparse lateral root formation. In stark contrast, *SiNHX7-OX* transgenic seedlings exhibited significantly longer primary roots and enhanced penetration into the media, indicating that *SiNHX7* overexpression alleviates the suppressive effects of salt stress on root growth. Quantitative analysis of primary root length (Figure 7B) further validated this observation, demonstrating significant differences between WT and *SiNHX7-OX* transgenic lines 10 days after transfer to plates. These findings confirm that *SiNHX7* overexpression promotes root growth under salt stress, facilitating improved nutrient and water uptake in saline environments.

Fresh and dry weight measurements are reliable indicators of biomass accumulation and plant vigor, directly reflecting salt tolerance capabilities. As shown in Figure 7C,D, *SiNHX7-OX* transgenic lines exhibited significantly higher fresh and dry weights than WT plants 10 days after transfer to treatment plates. These results indicate that *SiNHX7* overexpression promotes biomass accumulation under salt stress, enabling transgenic plants to maintain healthy growth and increase photosynthetic productivity, ultimately enhancing their overall salt tolerance.

Beyond root development, the overall salt tolerance phenotype provides key insights into plant resilience. As illustrated in Figure 7E and Supplementary Figure S3, three weeks of soil cultivation under salt stress conditions revealed striking phenotypic differences between WT and transgenic plants. WT plants exhibited severe growth inhibition and failed to bolt. In contrast, *SiNHX7-OX* transgenic lines maintained relatively healthy foliage and robust growth, although specific lines exhibited varying degrees of stress responses. For example, line *7aOX-1#* bolted normally but showed delayed pod development, while line *7aOX-5#* experienced slightly delayed bolting. Lines *7bOX-3#* and *7bOX-7#* bolted successfully but suffered from inflorescence wilting and delayed bolting, respectively. Collectively, these observations demonstrate that *SiNHX7* overexpression enhances the salt tolerance of transgenic *Arabidopsis* in soil environments.

## 3. Discussion

In this study, we identified nine *SiNHX* family members through a series of bioinformatics analyses, all of which contain the typical Na^+^/H^+^ antiporter domain (PF00999). These members exhibit high sequence homology, indicating that the family has retained conserved ion transport functions during evolution. Phylogenetic tree analysis revealed divergent evolutionary clades among family members (Figure 1), suggesting that functional differentiation may have occurred during long-term evolution to adapt to different environmental stresses or tissue-specific requirements. This coexistence of structural conservation and evolutionary divergence provides important insights into understanding the functional diversity of the *SiNHX* family in plant growth, development, and stress responses. Additionally, different *SiNHX* family members may exhibit distinct tissue expression patterns: most members are likely specifically expressed in roots, likely specializing in regulating root responses to salt stress, while certain members show higher expression in leaves and may participate in maintaining ion homeostasis in leaf cells (Figure 5). Furthermore, family members may play different roles in regulating salt tolerance across varying salt stress intensities and developmental stages. This functional specificity allows the *SiNHX* family to finely regulate plant responses to salt stress through synergistic interactions among members.

Previous studies have shown the impact of plant Na^+^/H^+^ antiporter genes on salt tolerance. For example, overexpression of *GmNHX6* in *Arabidopsis* maintains a low Na^+^/K^+^ ratio under saline–alkali conditions and enhances salt tolerance [40]. In rice, overexpression of *HtNHX1* or *HtNHX2* improves salt tolerance, and *HtNHX2*-transgenic rice maintains high yields under low-potassium or nutrient-deficient conditions, demonstrating significant application potential [39]. Our experimental results on *SiNHX7* show that overexpression of its two transcripts significantly enhances *Arabidopsis* salt tolerance, consistent with the conserved ion compartmentalization function of the *SiNHX* family. As a vacuolar membrane Na^+^/H^+^ antiporter, SiNHX7 reduces Na^+^ toxicity to cellular metabolism by transporting excess cytoplasmic Na^+^ into vacuoles, maintaining intracellular ion homeostasis and osmotic balance, thereby promoting seed germination, root growth, and overall biomass accumulation (Figure 7). Our findings are consistent with previous studies on gene overexpression, suggesting that Na^+^/H^+^ antiporters may share conserved salt tolerance regulatory mechanisms across plant species. However, Na^+^/H^+^ antiporter genes in different plants may differ in expression patterns, subcellular localization, and functional characteristics. The SiNHX7 protein likely possesses unique regulatory networks and functional traits that enable it to play a specific role in *Arabidopsis* salt tolerance.

Roots are critical organs for plant water and nutrient uptake, and their growth under salt stress directly affects plant survival and development. Our results show that *SiNHX7-OX* transgenic *Arabidopsis* seedlings exhibited significantly better root growth than WT when vertically grown on NaCl-containing medium (Figure 7A), with longer primary roots and more effective medium penetration, further confirmed by quantitative analysis (Figure 7B). This root growth advantage may arise from multiple factors. On one hand, as a vacuolar membrane Na^+^/H^+^ antiporter, SiNHX7 sequesters excess cytoplasmic Na^+^ into vacuoles, reducing Na^+^ toxicity to root cells and preserving root cells’ structural and functional integrity. This maintains normal root cell activities, including division, elongation, and differentiation, thereby promoting root growth. On the other hand, *SiNHX7* overexpression may influence root hormone metabolism and signaling. Plant hormones regulate growth and establish defense systems through signaling to help plants resist abiotic stresses [49]. *SiNHX7* overexpression may synergistically promote root growth under salt stress by affecting hormone levels and signaling pathways.

After salt stress treatment in soil, *SiNHX7-OX* transgenic *Arabidopsis* plants showed significantly superior overall salt tolerance phenotypes compared to WT (Figure 7C and Supplementary Figure S3). WT plants exhibited severe salt damage symptoms such as slow growth and failed bolting, while *SiNHX7-OX* transgenic plants generally maintained better growth, with some lines bolting normally despite interline variation. The improved overall salt tolerance phenotype results from the synergistic action of multiple organs and physiological processes. First, robust root growth provides adequate water and nutrient supply to the shoot system. The developed root system of *SiNHX7-OX* transgenic *Arabidopsis* efficiently absorbs water and nutrients from saline soil, meeting growth needs and alleviating shoot stress effects. Second, as the primary photosynthetic organs, leaf physiological status directly impacts plant growth. *SiNHX7* overexpression likely preserves chloroplast structure and function by maintaining ion and osmotic balance in leaf cells, ensuring normal photosynthesis. Additionally, *SiNHX7* overexpression may regulate stomatal movement, gas exchange, and water loss, further enhancing plant survival under salt stress.

During reproductive growth, different *SiNHX7-OX* transgenic lines exhibited varying salt tolerance responses. Line *7aOX-1#* bolted normally but showed delayed pod development under salt treatment; line *7aOX-5#* had slightly delayed bolting; line *7bOX-3#* bolted normally but suffered inflorescence wilting; and line *7bOX-7#* exhibited delayed bolting (Figure 7). These interline differences may arise from gene expression levels, genetic background, and environmental interactions. Variations in *SiNHX7* expression among lines likely contribute to differing salt tolerance capacities. Moreover, different transgenic insertion sites may affect neighboring gene expression, influencing salt tolerance phenotypes. Previous studies have shown that NHX proteins regulate not only salt tolerance but also plant growth and development. *Arabidopsis nhx5 nhx6* double knockout mutants exhibit low vacuolar pH in roots, impairing transmembrane protein sorting and vacuolar pH homeostasis, leading to small rosette leaves, dwarfism, delayed flowering, and low seed yield [21]. *Arabidopsis nhx1nhx2* mutants show retarded growth, with vacuolar pH and K^+^ content lower than WT [22,41]. Our results indirectly confirm that *NHXs* may participate in regulating *Arabidopsis* growth and development. Ideally, stress-resistant and high-yield crops are desirable, but balancing the growth–stress trade-off remains a huge challenge [50].

While this study has generated meaningful results, several limitations remain. First, our research focused exclusively on *SiNHX7* overexpression in *Arabidopsis*; as a model plant, these findings need validation and extension in crop species to assess their agricultural application potential. Second, the specific molecular mechanisms underlying *SiNHX7*-mediated salt tolerance—particularly its interactions with other genes and signaling pathways—require further investigation. Proteomics and transcriptomics approaches could comprehensively analyze the molecular network affected by *SiNHX7* overexpression, unraveling the regulatory mechanisms of salt tolerance.

## 4. Materials and Methods

### 4.1. Plant Materials and Growth Conditions

The gene expression profiles and functional characteristics of the foxtail millet cultivar Jingu21 (JG21) and *Arabidopsis* Col-0 were analyzed. Foxtail millet material JG21 was planted in a controlled environment chamber with a temperature of 25 °C and a 16 h light/8 h dark cycle. The foxtail millet material was grown in the greenhouse of the College of Agriculture, Shanxi Agricultural University. For tissue expression analysis, the roots, leaves, and whole seedlings of 7-day-old seedlings and the panicles and stems of 2-month-old seedlings were rapidly frozen in liquid nitrogen in a 2 mL centrifuge tube and then stored in a −80 °C refrigerator. There were at least three replicates of each material sampled for each experimental treatment. For induction expression analysis, 7-day-old seedlings were treated with 150 mM NaCl for 0 h, 1 h, 3 h, 6 h, 12 h, and 24 h, respectively. After treatment, whole seedlings were collected, quickly frozen in liquid nitrogen, and stored in a −80 °C refrigerator. There were at least three replicates of each material sampled for each experimental treatment.

### 4.2. Nomenclature of NHX Gene Family Members in Foxtail Millet

The complete genome sequence, the protein sequences, and the gene structure annotations of foxtail millet were sourced from Phytozome (https://phytozome-next.jgi.doe.gov/, accessed on 18 July 2024). The domain data from the Hidden Markov Model (HMM) PF00999 of the *NHX* gene family were retrieved from the Pfam Data website (http://pfam-legacy.xfam.org/, accessed on 18 July 2024). By utilizing TBtools (v2.056) [51], the conserved protein sequences of the *NHX* gene family in foxtail millet were extracted and compared to those of the *NHX* gene family in *Arabidopsis*, resulting in the identification of 9 *NHX* gene family members in foxtail millet.

### 4.3. Construct the Phylogenetic Tree of the NHX Gene Family

Multiple alignment of NHX protein sequences from foxtail millet, *Arabidopsis*, and rice was conducted using Clustal W. The evolutionary tree of the *NHX* gene family was constructed with the Neighbor Joining (NJ) method in MEGA 11, utilizing a Bootstrap parameter of 1000. All other parameters were set to their default values.

### 4.4. Examination of Physicochemical Characteristics of NHX Gene Family Proteins in Foxtail Millet

The physicochemical properties of *SiNHX* gene family proteins were analyzed utilizing TBtools, while the secondary structures of proteins were forecasted through the Cell PLoc 2.0 website (http://www.csbio.sjtu.edu.cn/bioinf/Cell-PLoc-2/, accessed on 18 July 2024).

### 4.5. Chromosome Mapping, Gene Structure, and Conserved Domain Analysis of the SiNHX Gene

The annotation file of the *SiNHX* gene structure obtained from the Phytozome website was visualized via chromosome mapping and gene structure analysis through TBtools. Conserved protein motifs of the *SiNHX* gene family were analyzed using the MEME online tool (http://meme-suite.org/tools/meme, accessed on 18 July 2024), and sequence conservation properties were visualized via TBtools based motif analysis.

### 4.6. Prediction of Cis-Acting Elements in the SiNHX Gene Promoter

The genome sequence of *SiNHXs* was obtained from the Phytozome website. Subsequently, the 2000 bp promoter sequence upstream of the foxtail millet *NHX* gene was extracted by TBtools. These promoter sequences were then submitted to the PlantCARE online platform (http://bioinformatics.psb.ugent.be/webtools/plantcare/html/, accessed on 21 November 2024) for the prediction of promoter cis-regulatory elements. Finally, visualization analysis of the results was conducted using TBtools.

### 4.7. Collinearity Analysis of the NHX Gene Family in Foxtail Millet

Using default parameters in TBtools, the collinearity relationships among *Setaria italica* (foxtail millet), *Oryza sativa* (rice), and *Arabidopsis NHX* genes were analyzed. Collinear gene pairs between foxtail millet and rice and foxtail millet and *Arabidopsis* were obtained and then visualized by TBtools.

### 4.8. Construction and Genetic Transformation of Gene Overexpression Vector

In order to construct *SiNHX7a-OE* and *SiNHX7b-OE* vectors, the coding sequences of *SiNHX7a* and *SiNHX7b* were amplified by specific primers. Then, KpnI and XbaI restriction enzymes were used to insert them downstream of the 35S promoter of the binary vector *p35S-MCS-EGFP-6×His-35S-Hyg*. The primers used in this experiment are shown in Supplementary Table S2. Overexpression vectors *35S::SiNHX7a-eGFP* and *35S::SiNHX7b-eGFP* were constructed utilizing a seamless cloning reaction system and introduced into *Agrobacterium tumefaciense* GV3101 receptor cells. *Agrobacterium*-mediated floral dip transformation was performed on Col-0 plants. *Arabidopsis* lines containing Col-0, as well as those overexpressing *SiNHX7a* and *SiNHX7b*, were cultivated in a controlled environment chamber at 21 °C under light conditions of 100 μmol m^−2^s^−1^, with a light cycle of 16 h light/8 h dark. T3 seeds were produced from the positive transgenic plants. For further analysis, we quantitatively characterized the transgenic lines using RT-qPCR (Supplementary Figure S4). Ultimately, the *35S::SiNHX7a-eGFP* transgenic lines *7aOX-1#* and *7aOX-5#*, along with the *35S::SiNHX7b-eGFP* transgenic lines *7bOX-3#* and *7bOX-7#*, were selected for subsequent experiments.

### 4.9. RNA Extraction, cDNA Synthesis, and RT-qPCR

RNA was extracted from plant materials using the FlaPure Plant Total RNA Extraction Kit (Genesand Biotech, Beijing, China), and subsequently reverse-transcribed into cDNA with the Union Script First-strand cDNA Synthesis kit (Genesand Biotech, Beijing, China). Gene expression levels were assessed using gene-specific primers (Supplementary Table S2) and SYBR Green Super Mix (Mei5bio, Beijing, China) on Bio-Rad CFX Duet (Bio-Rad CFX Duet, BIORAD, Hercules, CA, USA). A 20 µL RT-qPCR reaction mixture was prepared using 2 µL of cDNA obtained by reverse transcription as the template, which included 10 µL of 2 × Real-time PCR Supermix (SYBR Green, with anti-Taq), 0.5 µL each of 10 µM forward and reverse primers, and ddH_2_O to adjust the volume. The procedure was as follows: 95 °C for 1 min; 40 cycles were repeated at 95 °C for 15 s, 60 °C for 15 s, and 72 °C for 30 s. The foxtail millet actin gene *SiActin* (*Seita.8G043100*) was used as the internal reference [52]. The relative expression levels of target genes were calculated by 2^−ΔΔCT^.

### 4.10. Analysis of Seed Germination Rate

Mature seeds of *Arabidopsis* were collected and subsequently dried at room temperature for three weeks in preparation for germination experiments. Prior to the initiation of the experiments, the seeds underwent a sterilization process and were plated on 1/2 MS medium without or with a specified concentration of NaCl. After stratification at 4 °C in the dark for 2 days, the plates were transferred to an incubator maintained at 21 °C under a 16 h light/8 h dark cycle. Germination rates were analyzed from Day 0 to Day 7 after the plates were transferred to the light condition [53].

### 4.11. Salt Treatment of Transgenic Arabidopsis

Seeds of the *Arabidopsis* overexpression lines were sown in nutrient soil within a controlled environment chamber. Three-week-old *Arabidopsis* plants were then irrigated with a 200 mM NaCl solution for 15 days [54].

### 4.12. Statistical Analysis

The analysis of the data was conducted using one-way or two-way ANOVA, followed by Turkey’s multiple comparisons test. The significance levels were indicated as follows: ns: no significance; * *p* < 0.05; ** *p* < 0.01; *** *p* < 0.001; **** *p* < 0.0001; or lowercase letters at *p* < 0.05.

## 5. Conclusions

In conclusion, this study systematically analyzed nine *SiNHX* genes in foxtail millet (*Setaria italica*) through bioinformatics, revealing the structural, regulatory, and transcriptional characteristics of this family. We demonstrated that overexpression of both long and short transcripts of the *SiNHX7* gene significantly enhances salt tolerance in transgenic *Arabidopsis*. These findings provide new insights into the functions and evolution of plant Na^+^/H^+^ antiporter families, offer a theoretical basis for understanding plant salt tolerance mechanisms, and lay a foundation for using genetic engineering to develop salt-tolerant crop varieties. With further research, the application potential of the *SiNHX7* gene is expected to be further exploited, providing effective solutions to mitigate the constraints of saline soil on agricultural production.

## Figures and Tables

**Table 1:** Basic physicochemical properties of SiNHX proteins in foxtail millet.

Gene Name	Gene ID	Molecular Weight	Theoretical pI	Instability Index	Aliphatic Index	Grand Average of Hydropathicity
*SiNHX1*	Seita.2G160100.1	26,780.57	5.26	49.12	115.37	0.821
*SiNHX2*	Seita.2G160200.1	32,416.94	5.31	45.86	84.56	0.062
*SiNHX3*	Seita.2G249400.1	53,875.11	5.81	40.57	102.49	0.367
*SiNHX4*	Seita.2G422800.1	59,209.59	8.63	36.27	110.65	0.588
*SiNHX5*	Seita.3G038800.1	59,818.53	7.68	31.02	115.68	0.645
*SiNHX6*	Seita.3G409000.1	128,696.73	6.65	43.34	102.63	0.052
*SiNHX7*	Seita.4G138500.1	58,178.09	8.37	31.47	107.43	0.605
*SiNHX8*	Seita.7G006000.1	21,103.35	9.87	46.25	64.54	−0.514
*SiNHX9*	Seita.8G215400.1	59,596.85	8.99	38.46	111.41	0.548

**Table 2:** Prediction of secondary structure and subcellular localization of SiNHX proteins.

Gene Name	Gene ID	Alpha Helix (%)	Extended Strand (%)	Beta Turn (%)	Random Coil (%)	Subcellular Localization
*SiNHX1*	Seita.2G160100.1	47.97	17.89	0	34.15	Vacuole
*SiNHX2*	Seita.2G160200.1	54.01	12.54	0	33.45	Vacuole
*SiNHX3*	Seita.2G249400.1	51.21	12.15	0	36.64	Vacuole
*SiNHX4*	Seita.2G422800.1	48.98	12.80	0	38.22	Vacuole
*SiNHX5*	Seita.3G038800.1	53.66	12.82	0	33.52	Vacuole
*SiNHX6*	Seita.3G409000.1	55.17	9.40	0	35.43	Cell membrane
*SiNHX7*	Seita.4G138500.1	49.43	13.02	0	37.55	Vacuole
*SiNHX8*	Seita.7G006000.1	40.98	15.85	0	43.17	Vacuole
*SiNHX9*	Seita.8G215400.1	45.70	12.98	0	41.32	Vacuole

## Data Availability

The authors declare that the data supporting the findings of this study are available within the paper and its Supplementary Information Files. Raw data files in alternative formats may be obtained from the corresponding author upon reasonable request.

## Supplementary Materials

The following supporting information can be downloaded at: https://www.mdpi.com/article/10.3390/ijms26157139/s1.
