# Supplementary material for: Genome-Wide Identification of the SiNHX Gene Family in Foxtail Millet (Setaria Italica) and Functional Characterization of SiNHX7 in Arabidopsis
Source: Int J Mol Sci. 2025 Jul 24;26(15):7139. doi: 10.3390/ijms26157139 (PMC12345724; doi:10.3390/ijms26157139)
Supplement: Supplementary file 1 [file ijms-26-07139-s001.zip › ijms-3729555-supplementary.pdf]

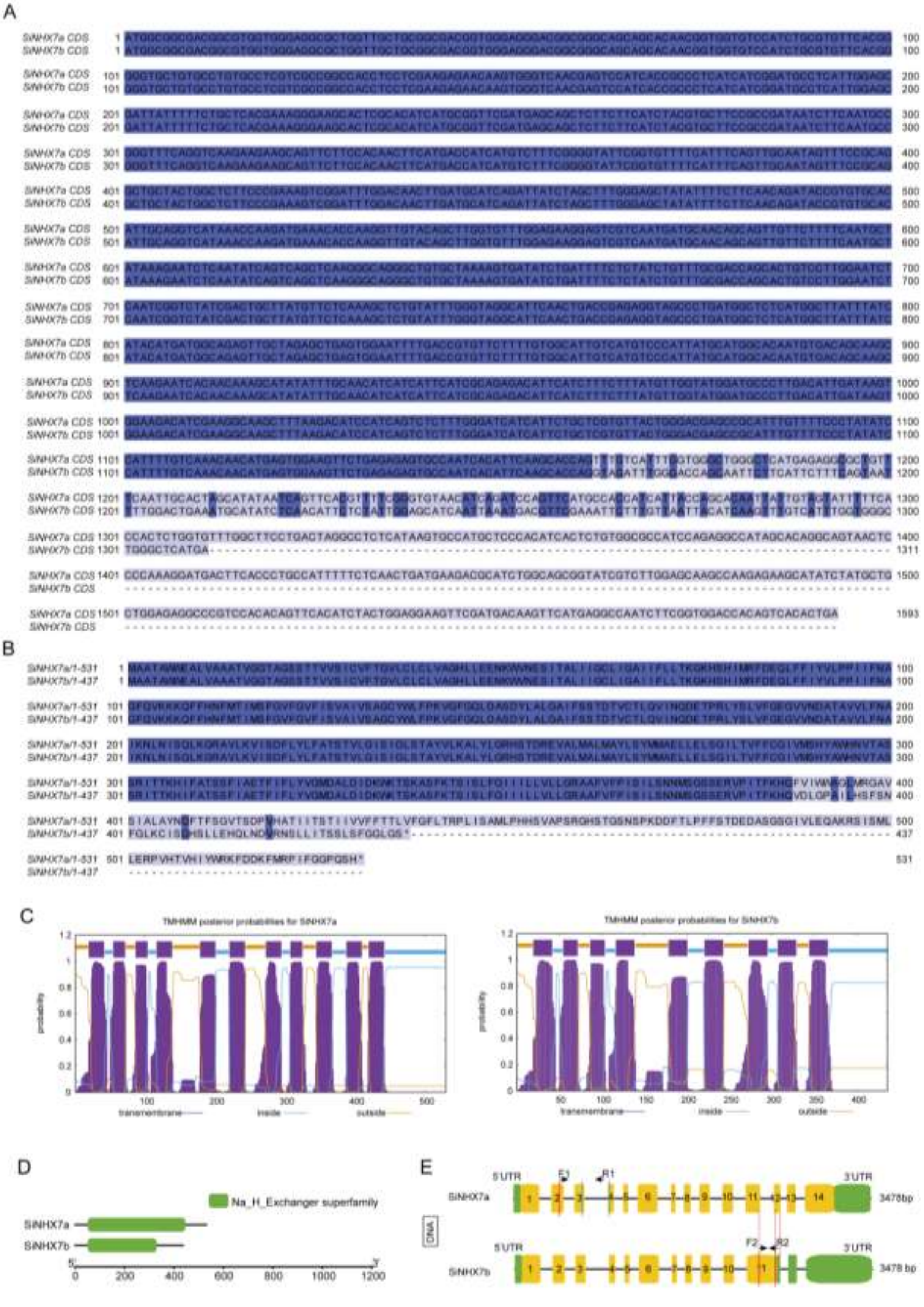

Supplementary Figure S1. Sequence alignment of two SiNHX7 transcripts and analysis of encoded proteins.

(A) CDS sequence alignment of two transcripts.

(B) Sequence alignment of amino acids encoded by *SiNHX7a* and *SiNHX7b*.

(C) Coding sequences of *SiNHX7a* and *SiNHX7b* are subjected to the TMHMM-2.0 website for transmembrane domain prediction.

(D) Functional domains of SiNHX7a and SiNHX7b.

(E) The schematic diagram illustrates the design of specific primers for distinguishing between two transcripts of *SiNHX7*. Yellow boxes and numbers represent exons, black solid lines represent introns, and arrows indicate the locations where the RT-qPCR primers were designed. Given that the CDS sequence of *SiNHX7a* corresponds to a subset of the *SiNHX7b* CDS sequence, we designed specific RT-qPCR primers exclusively for *SiNHX7b*.

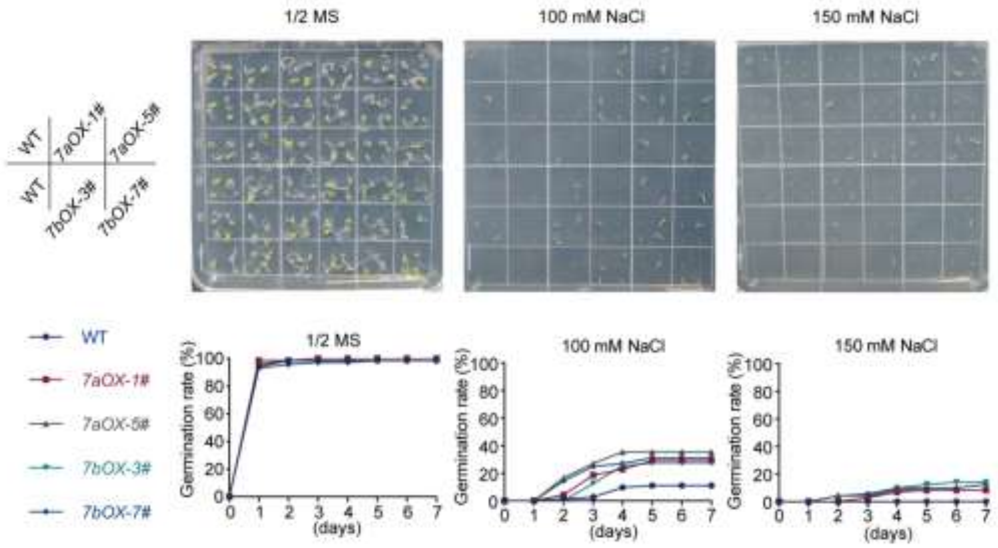

Supplementary Figure S2. Seed germination phenotypes and germination rates of wild-type (WT) and *SiNHX7-OX* seedlings on 1/2 MS medium with or without NaCl. Scale bar = 1 cm.

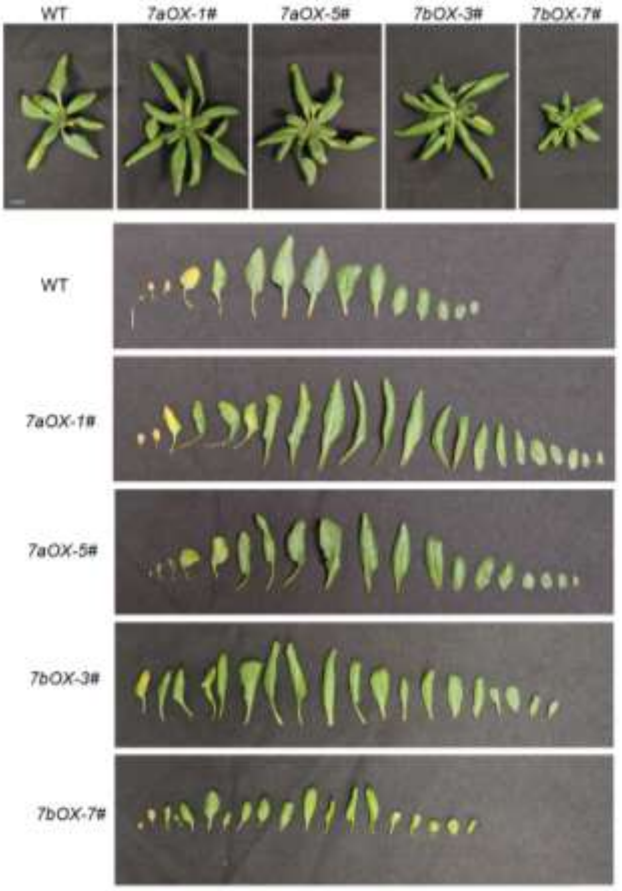

Supplementary Figure S3. Phenotypic characteristics of three-week-old wild-type (WT) and *SiNHX7-OX* *Arabidopsis* plants. Scale bar = 1 cm.

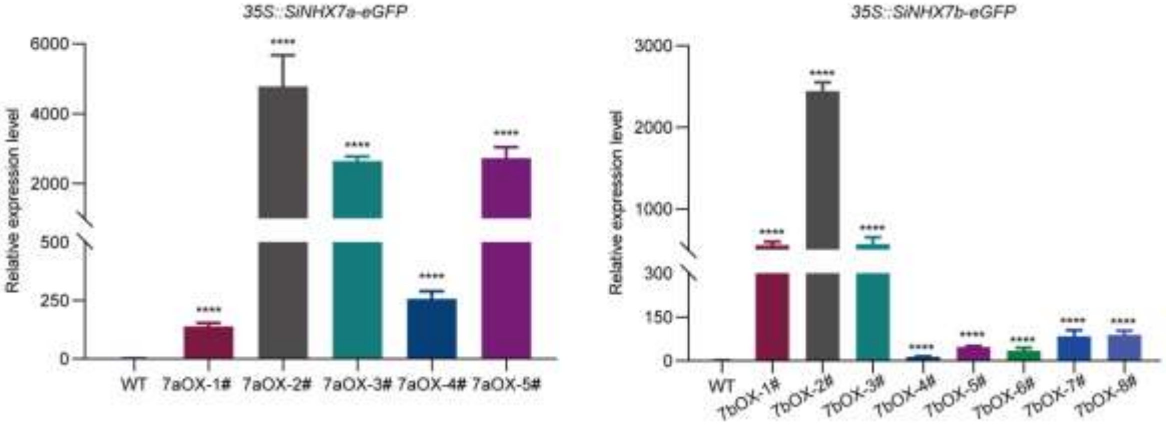

Supplementary Figure S4. Detection of expression levels of WT (Col-0) and *35S::SiNHX7-eGFP* transgenic lines through RT-qPCR. Data represent means ± SD (n=3). Significant difference is analyzed based on unpaired t test, **** $p < 0.0001$.

Supplementary Table S1 The Gene ID of *NHX* genes in three species

Species	Gene Name	Gene ID
Arabidopsis thaliana	*AtNHX1*	AT5G27150.1
	AtNHX2	AT3G05030.1
	AtNHX3	AT5G55470.1
	AtNHX4	AT3G06370.1
	AtNHX5	AT1G54370.1
	AtNHX6	AT1G79610.1
	AtNHX7	AT2G01980.1
	AtNHX8	AT1G14660.1
Oryza sativa	*OsNHX1*	LOC_Os07g47100.2
	OsNHX2	LOC_Os11g42790.1
	OsNHX3	LOC_Os05g05590.1
	OsNHX4	LOC_Os06g21360.1
	OsNHX5	LOC_Os09g11450.1
	OsNHX6	LOC_Os09g30446.1
	OsNHX7	LOC_Os12g44360.2
Setaria italica	*SiNHX1*	Seita.2G160100.1
	SiNHX2	Seita.2G160200.1
	SiNHX3	Seita.2G249400.1
	SiNHX4	Seita.2G422800.1
	SiNHX5	Seita.3G038800.1
	SiNHX6	Seita.3G409000.1
	SiNHX7	Seita.4G138500.1
	SiNHX8	Seita.7G006000.1
	SiNHX9	Seita.8G215400.1

Supplementary Table S2 Primers for foxtail millet gene

Gene name	Primer name	Function	Primer sequence (5'-3')
SETIT_026509mg	SiActin	RT-qPCR	F: CAGGGAGAAGATGACCCAAATC<br>R: CACCAGAGTCCAGCACAATAC
Seita.2G160100.1	SiNHX1		F: TTCTACTACCTCCCCGAGGC<br>R: ACGTCCCAAGAATGGCGAAA
Seita.2G160200.1	SiNHX2		F: CCCTTGACCACTCGTTACCT<br>R: GCGCTGCGAATTGTCTGAAA
Seita.2G249400.1	SiNHX3		F: CTTCAGATATCGATGCTCGTGCT<br>R: TTTTGGTGCTAAGCTGGTGC
Seita.2G422800.1	SiNHX4		F: TGCTGGCTGAGTTGCTAGAC<br>R: GATTTTCCGGGGCTGTCACT
Seita.3G038800.1	SiNHX5		F: AGCATCTGGTCATACTGCGG<br>R: TGGCGCGGTGAGAATAAACT
Seita.3G409000.1	SiNHX6		F: GTGCTGCGTGTGCTAAAGAC<br>R: TGCGCCTACCAAAGGATGAG
Seita.4G138500.1	SiNHX7a		F: CATCATGCGGTTCGATGAGC<br>R: GTAGCAGCCTGCGGAAACTA
Seita.4G138500.2	SiNHX7b		F: AGCACCAGGTAGATTTGGGAC<br>R: CCAGCCCACCAAATGACAAA
Seita.7G006000.1	SiNHX8		F: CTCCCACCAAACGCAGAGG<br>R: GCTTGGTTGTCACTCTTGAGC
Seita.8G215400.1	SiNHX9		F: GCTGTGTCCATTGCGTTAGC<br>R: CGCTTGGTTCCCTGGATAGG
Seita.4G138500.1	SiNHX7a	Construction of gene overexpression vector	F: ACGGGGGACGAGCTCGGTACC ATGGCGGCGACGGCGTG<br>R: TGGCGCGCCGGGCCCTCTAGA GTGTGACTGTGGTCCACCGAAG
Seita.4G138500.2	SiNHX7b		F: ACGGGGGACGAGCTCGGTACC ATGGCGGCGACGGCGTG<br>R: TGGCGCGCCGGGCCCTCTAGA TGAGCCCAGCCCACCAAATGAC
